# Development of Some Larval Nematodes in Experimental and Natural Animal Hosts: An Insight into Development of Pathological Lesions vis-a-vis Host-Parasite Interactions

**DOI:** 10.1155/2013/162538

**Published:** 2013-12-23

**Authors:** N. Chowdhury, N. K. Sood, Shyam Lal, Kuldip Gupta, L. D. Singla

**Affiliations:** ^1^Department of Veterinary Parasitology, College of Veterinary Science, Guru Angad Dev Veterinary and Animal Sciences University, Ludhiana, Punjab 141004, India; ^2^Department of Veterinary Pathology, College of Veterinary Science, Guru Angad Dev Veterinary and Animal Sciences University, Ludhiana, Punjab 141004, India

## Abstract

Infective third-stage larvae of three spiruroid nematodes, *Ascarops strongylina* and *Physocephalus sexalatus* of pigs and *Spirocerca lupi* of dogs, were recovered from 14 species of coprophagous beetles belonging to 4 different genera. These larvae were fed to rabbits and/or guinea pigs to study their development in these experimental hosts. Larvae of *A. strongylina* reached the adult stage in all rabbits and one guinea pig. The adult worms recovered in these hosts were 40% and 4%, respectively, and became diminutive in comparison to their natural hosts. The larvae of *P. sexalatus* became reencysted in the gastric wall of rabbits inducing marked pathological changes. The infective larvae of *S. lupi* became reencapsulated in the stomach wall of the rabbit and also showed development in the aortic wall. Adults of *Toxocara canis* of dog, collected from 5 different regions of the Indian subcontinent, varied significantly in size. The mouse passage of infective larvae of one of these types led to the recovery of the adults from the experimental dogs that were smaller in size and caused severe pathology in natural experimental hosts. Developmental effects shown in experimental hosts and host specificity are of value in understanding the evolution of nematode parasitism.

## 1. Introduction

Host-parasite interactions by parasitism remained unexplained till 20th century. Graham Bell and Austin Bert from Canada hypothesized the term, “a recombination, favoured by antagonistic” coevolution between the host and the parasite [1]. It was further defined as the resultant product of ecological, sociological, and physiological causes [2]. The phenomenon not only increased the parasite's reproductive capacity but also enhanced its virulence and/or pathogenicity. Host specificity, on the other hand, is the result of coaccommodation between the two, that is, the host and the parasite. The nature of the parasite, ambient conditions, and the host infected are the key factors determining the outcome of such a relationship in terms of virulence, pathogenicity, reproductive potential, maturity of the parasite, and finally the response of the host. In this communication, experimental results on development of some larval nematodes in natural and experimental animal hosts have been discussed in connection to host-parasite relationship.

## 2. Materials and Methods

The infective third-stage larvae (L_3_) of the spiruroid nematodes (Table 1) were obtained from 14 species of naturally-infected coprophagous beetles belonging to 4 different genera. The larvae were studied under coverslip preparation and identified as described by Alicata [3], Porter [4], Watanabe [5], and Ryzhikov and Nazarova [6]. In all experiments, the infective larvae were administered with the help of a stomach tube to the experimental and natural hosts, namely, rabbit, guinea pig, mice, or pups. During this study, unidentified larvae recovered from the beetle hosts were fed to 2 rabbits.

The differently-developed juveniles/larvae collected from viscera of the animals were studied alive and from the preserved specimens after appropriate clearing. The tissues showing conspicious gross lesions were fixed in 10% formalin and serially-cut into 5-6 *μ*m thick paraffin sections and stained with haematoxylin and eosin for histopathological evaluation.

The female specimens of *Toxocara canis* collected either in physiological saline or 10% formalin (Tables 2 and 3) were obtained either locally from pups/dogs or were received by us from 4 different cities in Assam, Gujarat, M.P., and U.P. located at a distance of 800–2,500 kms from Ludhiana, Punjab, India

For mouse passage, 12 albino mice were administered 100 second stage larvae (Table 4) at fortnightly interval, for a period of 1.5 months. The deskinned mice cut into pieces, were fed to 8 pups/dogs (one mouse each) no earlier than two months postinfection. The animals were maintained as per Institutional Animal Ethics Committee Guidelines. The data were analysed using SPSS 16.0 version by one-way analysis of variance and the means were compared by Tukey's b and Duncan's Multiple Range Test.

## 3. Results

**Figure 1:**
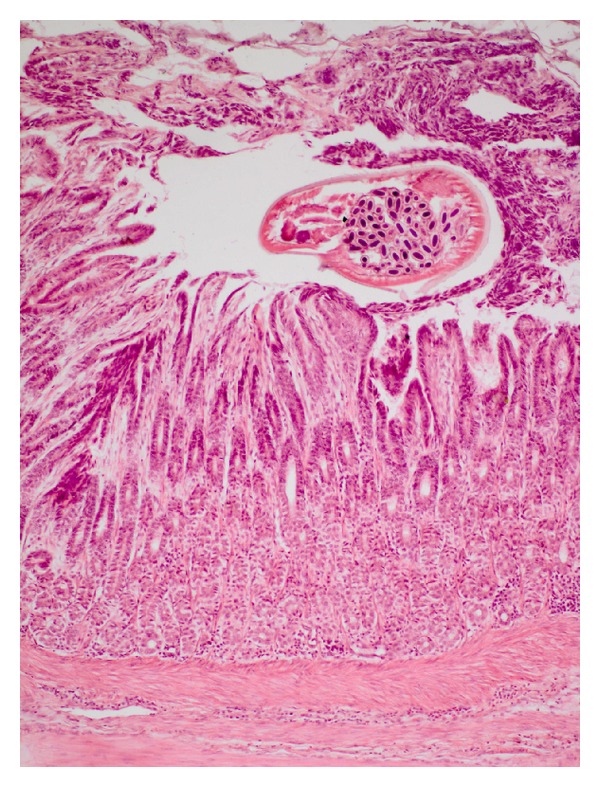
Photograph of stomach of rabbit showing a gravid female of *A. strongylina* cut transversely towards the lumen. Note denudation of superficial lining of epithelium (H. E. original magnification × 100X).

**Figure 2:**
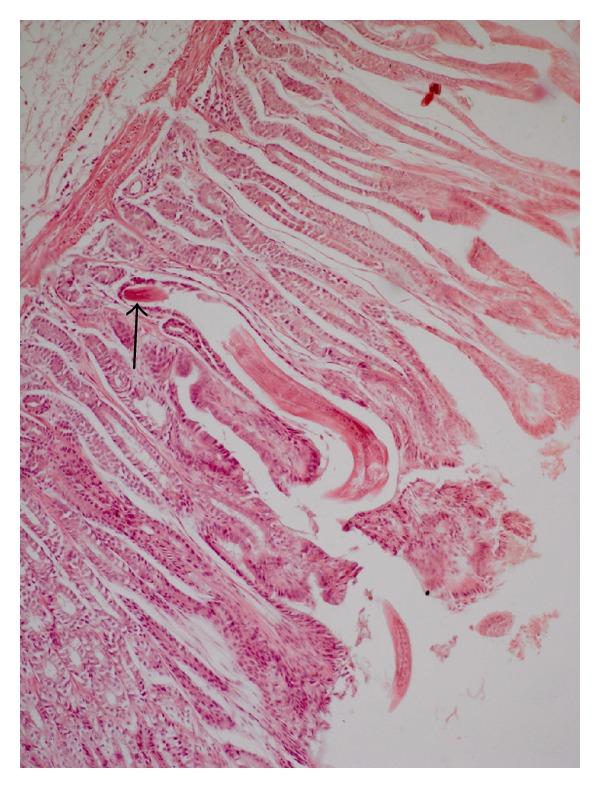
Section of stomach of rabbit showing a juvenile *A. strongylina* longitudinally cut deep into the mucosa. Arrow indicates head end of the L_4_ (H. E. original magnification × 100X).

The distinctive development patterns of the three spiruroid nematodes including two stomach worms of pig, that is, *Ascarops strongylina* and *Physocephalus sexalatus* and the third, the oesophageal worm of dog, that is, *Spirocerca lupi* are depicted in Table 1. The experimental infection of *A. strongylina* in rabbit and guinea pigs showed a marked difference in development and recovery of the parasite. Microscopically, the pathological lesions in *A. strongylina* consisted of damage by the adults and juveniles to the lining of mucosa and destruction of the underlying glands by the young adults that reached nearly the base of the mucosa (Figures 1 and 2).

**Figure 3:**
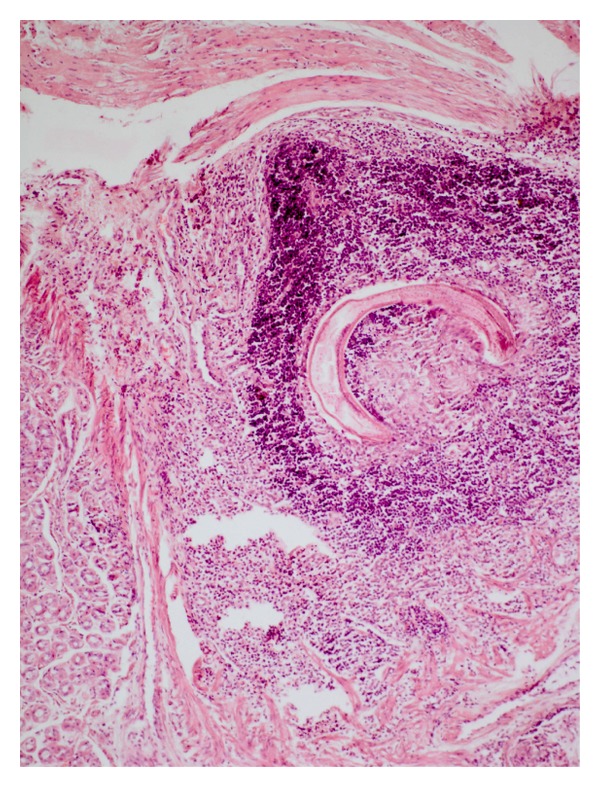
Section of stomach of a rabbit with the longitudinally cut larva of *P. sexalatus* in the submucosa; parasite is surrounded by a large rim of inflammatory cells-lymphocytes, eosinophils, and connective tissue (H. E. original magnification × 100X).

**Figure 4:**
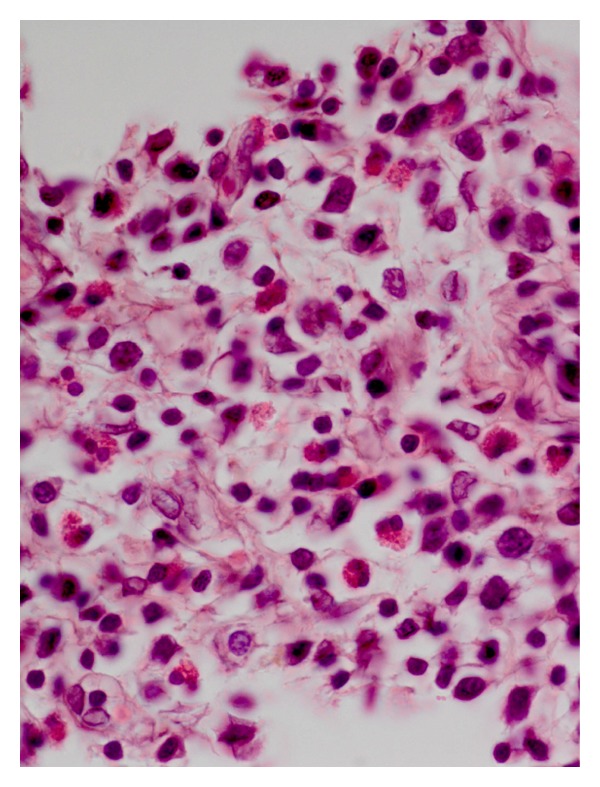
Higher magnification of Figure 3 showing more conspicuous eosinophils, macrophages, and a few fibroblasts among inflammatory cells (H. E. original magnification × 400X).

**Figure 5:**
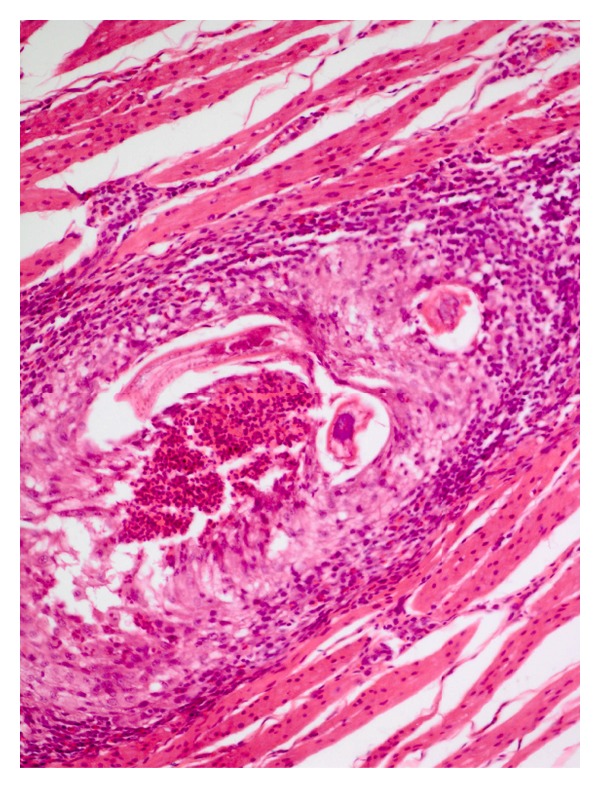
Parasitic granuloma in the tunica muscularis of stomach with chronic inflammatory cells including fibroblasts and cut sections of *P. sexalatus* surrounded by an adjacent zone of necrosis (H. E. original magnification × 100X).

In contrast to *A. strongylina*, *P. sexalatus* showed poor development and recovery in infected rabbits. In one rabbit, a third-stage larva was found that had migrated to the omentum (Table 1), whereas in two other animals, L_3_ remained arrested mostly in the gastric wall and encapsulated in different layers without undergoing further development. The encapsulated juveniles were found in the mucosal, submucosal, and muscular coats (Figures 3 and 5) of the gastric wall. The nodular lesions revealed the parasites surrounded by necrotic debris, neutrophils, and activated macrophages. At the periphery of such lesions, the inflammatory reaction consisted of lymphocytes, macrophages, and eosinophils, while in the areas adjacent to the mucosa, there were aggregates of macrophages and lymphocytes (Figure 4).

**Figure 6:**
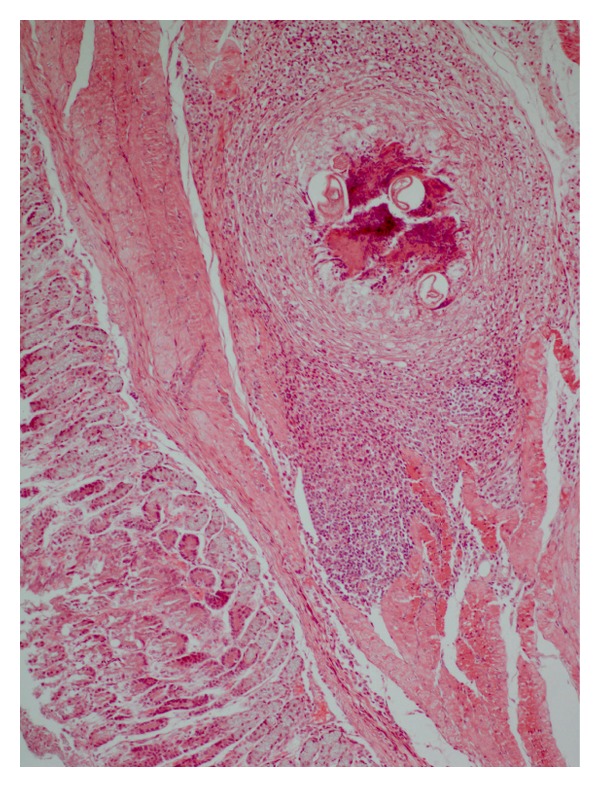
Sections of stomach showing an old parasitic granuloma of an unidentified larva and chronic inflammatory changes along with necrosis, fibrosis, and dystrophic calcification surrounded by a distinct fibrous encapsulation (H. E. original magnification × 100X).

The unidentified larvae did not develop in any of the rabbits, rather they became reencapsulted in the submucosa (Figure 6). Adjacent to the sections of this parasite, chronic inflammatory response consisted of necrosis and dystrophic calcification, surrounded by extensive fibroplasia and eosinophils.

In two experimental rabbits, the L_3_ of *S. lupi* were found, not only reencapsulated in the gastric wall, but some of the infective larvae were seen to have migrated to the aorta causing typical lesion of “aortic spirocercosis.” The recovered larvae from the gastric wall of the rabbits measured: 2.18 mm × 0.093 mm–2.52 mm × 0.097, which falls in the range of L_3_ recovered from the dung-beetles (2.19 mm × 0.085–2.48 mm × 0.12 mm). Interestingly, the young adults recovered from the aorta measured: 2.58 mm × 0.17–5.29 mm × 0.21 mm.

**Figure 7:**
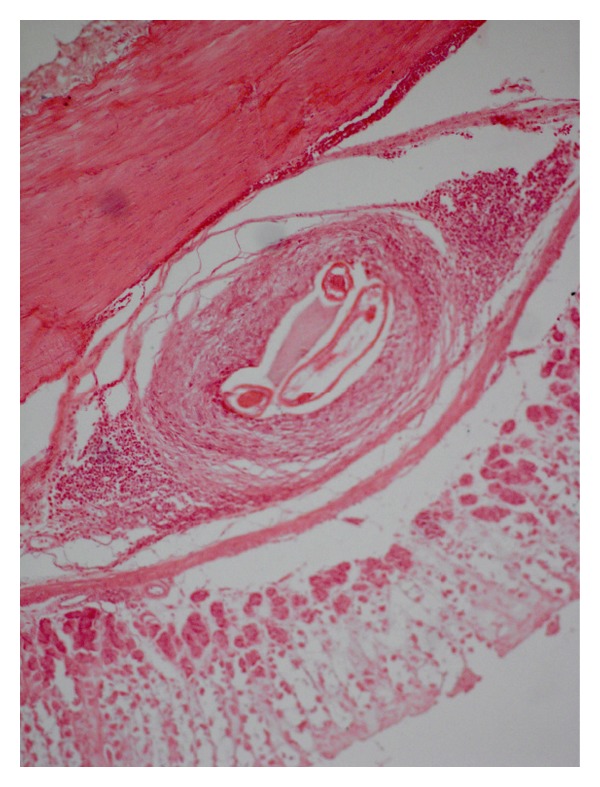
A section of stomach rabbit showing chronic granuloma around *S. lupi* in the submucosa with extensive fibrosis around the parasite and a peripheral zone of chronic inflammatory cells (H. E. original magnification × 100X).

The histopathology of the lesions from the stomach wall revealed oedema of the gastric mucosa and a zone of necrosis, surrounded by extensive inflammation, consisting predominantly of macrophages, lymphocytes, and some eosinophils. Early fibroblastic proliferation was also evident. Around the parasite in the submucosa, activated macrophages were found surrounded by lymphocytes, eosinophils, and mature collagen fibres (Figure 7).

**Figure 8:**
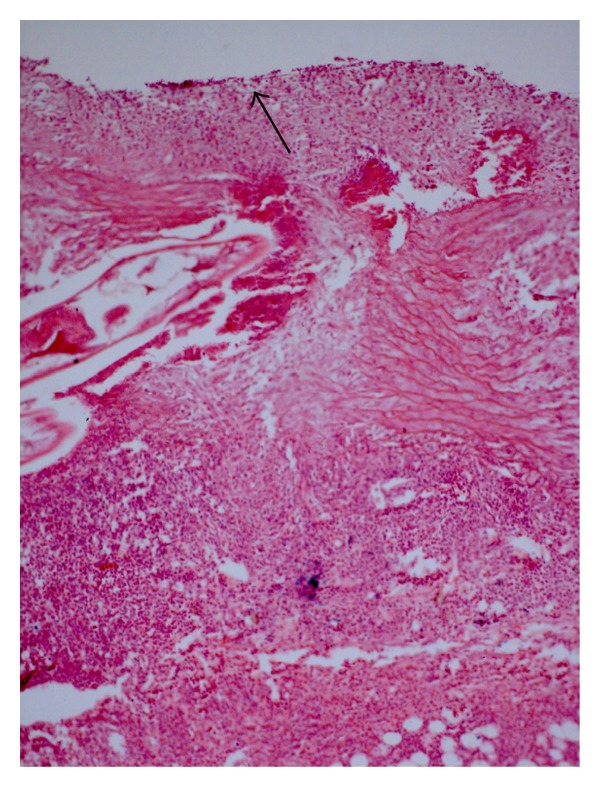
Section of aorta of a rabbit with a longitudinally cut section of *S. lupi* in media. Note the extensive tissue damage, necrotic debris in the parasitic tunnels/cavities, fragmented tunica elastica interna, and marked infiltration of inflammatory cells. Arrow indicates intima (H. E. original magnification × 100X).

**Figure 9:**
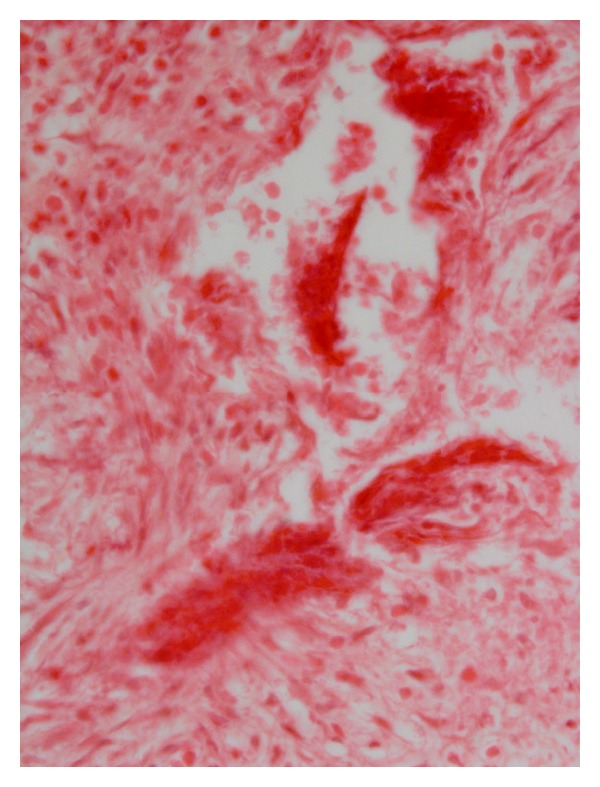
A section of chronically-infected aorta with *S. lupi* in the intimal region showing parasite remnants in dystrophic calcification surrounded by fibroblastic reaction within tunica intima (H. E. original magnification × 400X).

**Figure 10:**
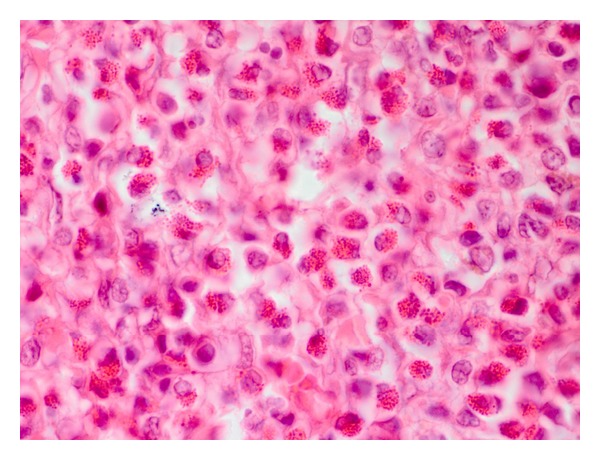
Section of another aorta infected with *S. lupi* depicting marked infiltration of eosinophils and plasma cells (× 400).

In the aorta, the three layers depicted varying grades of changes. The intima was greatly thickened owing to proliferation of fibro-cellular tissue (Figure 9). The endothelial lining was disrupted and internal elastic lamina was fragmented (Figure 8). In the parasitic tunnels, a hyalinised necrosed thin layer was surrounded by connective tissue proliferation and inflammatory response, predominantly consisting of lymphocytes and eosinophils, a few plasma cells, and macrophages. In the media, there was a marked infiltration of eosinophils, many plasma cells, some lymphocytes, and a few macrophages (Figure 10). In the outer media, serofibrinous exudation was observed, in addition to necrotic foci having dystrophic calcification particularly inside the tracts left by the juveniles (Figure 9). In the tunica adventitia, the changes were similar but relatively milder.

**Figure 11:**
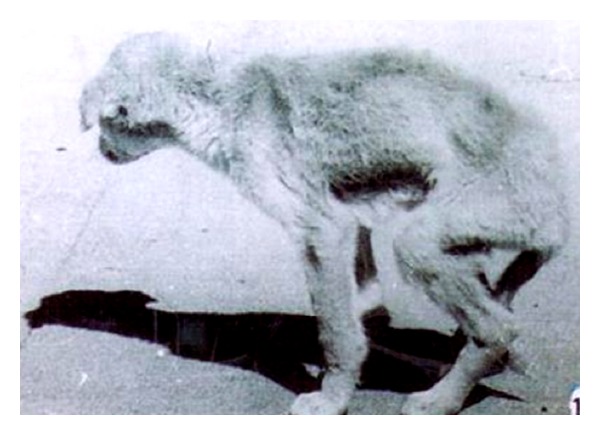
A highly emaciated infected dog with *T. canis*. Note the tucked up abdomen and posterior paralysis.

The adult gravid females of *T. canis* collected from 5 different regions of the Indian subcontinent were significantly different in length, diameter, and area (Tables 2 and 3). The experimental pups/dogs, which were fed L_2_ of *T. canis* via mouse passage (Table 4), exhibited a series of clinical signs, including diarrhea and debility (Figure 11).

**Figure 12:**
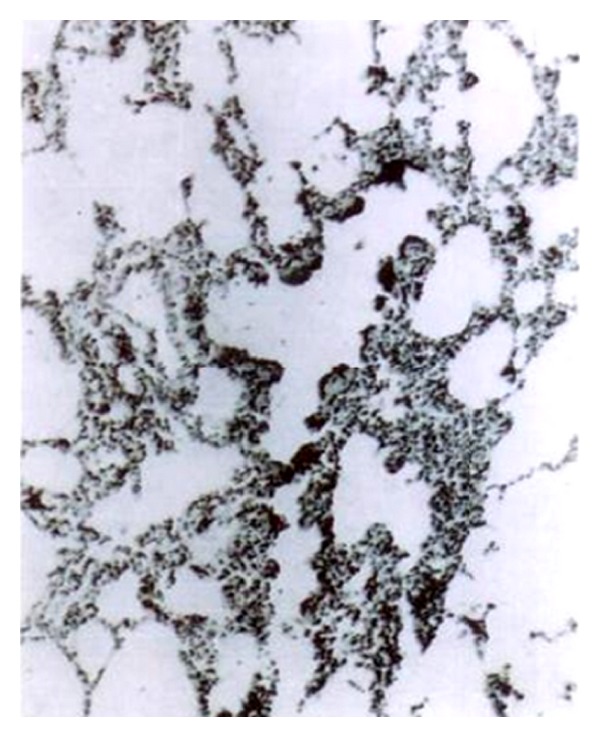
Section of lung of *T. canis*-infected dog depicting interstitial pneumonia (H. E. original magnification × 70X).

**Figure 13:**
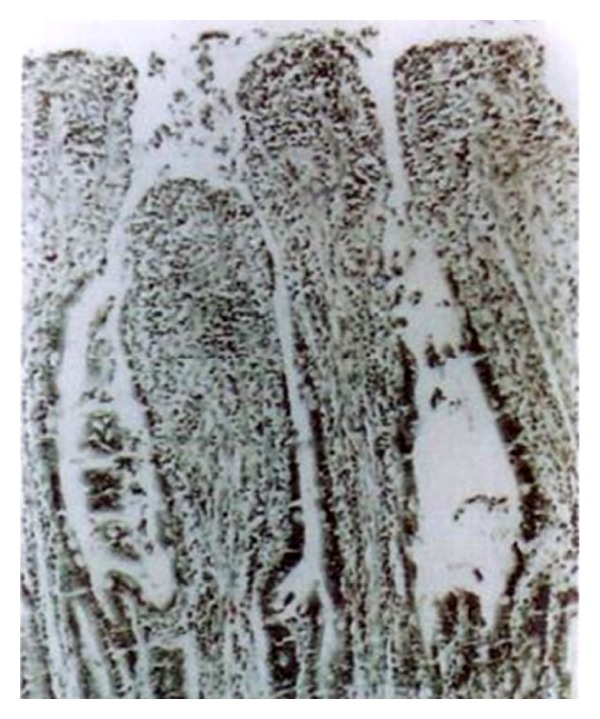
Section of intestine of the same dog showing necrotic enteritis and exfoliation of cells (H. E. original magnification × 70X).

On postmortem examination (26–89 DPI), the intestine revealed congestion, oedema, and consolidation in lungs. Microscopically, the liver showed portal hepatitis and vacuolar degeneration of hepatocytes, while the lung depicted interstitial pneumonia (Figure 12). The intestine revealed necrotic enteritis with marked infiltration of lymphocytes and plasma cells in the lamina propria leading to thickening and blunting of villi. There was exfoliation of the lining epithelium of the tips of villi (Figure 13). The crypts of Liberkuhnn showed globlet cells hyperplasia.

## 4. Discussion

Watanabe [5] was the first researcher who succeeded in infecting three rabbits with third-stage larvae of *A. stronglina* and the recovery percentages were 35%, 23.3%, and 20%, respectively. The adult worms were recovered between 40 and 112 DPI. In the present experiments in 5 rabbits, the recovery was from 36.0 to 45.3 percent, which was much higher in comparison to the earlier study [5]. The recovery of adults from one guinea pig in this study, however, was very low (4%) and the other guinea pig was negative (Table 1). The prepatent period of *A. stronglina* was found to be 41 days in rabbits. The adult worms recovered from the animals were diminutive in size than the natural host, the pig. From this experiment, it appeared that the rabbit was a good experimental model for *A. strongylina* infection. Previously, mature adults of *A. strongylina* were collected from wild rodents as second record as a definitive host of this nematode [7].

During experiment with *P. sexalatus* in three rabbits, the L_3_ of this parasite did not develop to adulthood but became reencapsuled in the gastric wall inducing severe pathological changes, though both parasites, that is, *A. strongylina* and *P. sexalatus* are known to reside in the stomach of the same natural host, that is, the pig. The recovery of an L_3_ from the omentum could simply be due to erratic migration or due to the effect of biological “incompatibility” between the parasite and its host to avoid host's immune response or due to a more primitive lifestyle it had pursued before it had coaccommodated in its natural host, the pig. Undoubtedly, physiology/ecology and other factors in the stomach of rabbit and pig are unlikely to be similar.

The experiments with the L_3_ of *S. lupi* revealed that in both rabbits, the larvae not only reencysted in the gastric wall, but also followed the migratory route to the aorta as in natural host that is, dog. In a recent study, ten beagle dogs were experimentally challenged with 40 infectious *S. lupi* larvae orally [8]. These dogs died due to rupture of an aortic aneurism. Seven dogs became infected and presented with esophageal nodules and worm eggs in their feces. One dog did not become infected [8].

The pathological changes induced by the larvae of *P. sexalatus* were rather chronic in nature in the gastric wall than those in the aorta. In the aortic wall the change was more acute, particularly in the tunica media. The development of *S. lupi* both in the gastric wall and its migration to the aortic wall are, therefore, interesting for both “parasitism” and “specificity” of the parasite-host system.

In Russia, ruminants, particularly sheep and cattle may act as normal definitive hosts of the two species, *A. strogylina* and *P. saxalatus*, whereas, in other parts of the world, these two species are found only in pigs [9]. Phylogenetically rabbit is a herbivore in contrast to guinea pig, which is akin to carnivorous host. Differential development of the two species of these helminth parasites in different regions of the world demonstrates that not only genetic characterization and the variability in their evolution affect their development, but also climatic and ecological factors of the region are responsible for the host specificity. The same holds largely true for *S. lupi*, which is the typical parasite of canines, namely, dog, fox, and jackal, although in the present experimental study, it showed partial development in a herbivore like rabbit.

We presume that significant differences in length, diameter, and area of adult gravid females of *T. canis* collected from 5 different regions of the Indian subcontinent might be related to the production of more eggs in *T. canis*. Sonin and Rykovoskii [9] also reported that the longer worms, Ascaris lumbricoides, produced more eggs. However, passage through mice of infective eggs from medium-sized worms (Assam type) and the eventually recovered worms from pups/dogs were found to be reduced in length, which were comparable to small form of local type (Table 3). The reduction in length, fecundity and prepatency is known to be influenced by several factors, namely, host's diet, age, size, and immune status, besides space and nutrient quality available in the gut [10–12]. This aspect was reviewed by Poulin [13].

After feeding infective larvae of *T. canis* via mouse passage, Herschel [14] determined the prepatent period in dogs to be 34–39 days, almost similar to what recorded in the present study. Most of the infected pups/dogs in the our study (Table 4) developed loss of appetite and diarrhea in early stages, followed by constipation, tucked up abdomen, extreme emaciation, loss of body weight, and gradual weakness in legs or posterior paralysis at later stage (Figure 11). Van Heerdan [15] observed muscular weakness in a dog (adult natural infection) associated with *T. canis* infection. In the present experiment in dogs, the inflammatory changes observed in the intestine resembled the findings of Litvishko [16], although the severity of lesions was more pronounced. Whether the pathological changes and clinical signs were due to the parasite's passage through mice, variation in the host species, or the strain of parasite is unclear. Zyngier and Santa Rosa [17] and Dunsmore et al. [18] suspected that there may be strain differences in *T. canis*. Our experiments in mice, rabbits, and monkeys demonstrated acute pathological changes in different organs. An interesting aspect of the study was that in the experimental hosts, the inflammatory response got exaggerated as compared to that reported in natural hosts. The altered pathological response in experimental host may be due to abnormal localization and/or poor adaptation to the tissue microenvironment of experimental/new hosts [13].

## 5. Conclusion

The present study employing several species of nematodes in natural and experimental hosts demonstrated significant variations in the development of the parasite, pathological responses, and host-parasite specificity, which may be of value in understanding the ecology and evolution of parasitism, particularly those having zoonotic importance, namely, *T. canis* and *S. lupi. *The study also throws light on the development of pathological lesions vis-a-vis host-parasite interactions.

## Figures and Tables

**Table 1:** Experimental infection of laboratory animals with spiruroid nematodes.

Experimental animal	Infective dose (L_3_)	Mode of oral infection	Necropsy (DPI)	Results	Recovery percentage	Remarks
*As* *ca* *ro* *ps* *strongylina*						
R_1_	75	Single dose	22	M = 5 (+YA 3) F = 17 (+YA 2)	36.0	Several worms (M or F) were under the 4th ecdysis
R_2_	121	Single dose	48	M = 24 (+YA 2) F = 24	41.5	
R_3_	100	2 successive days (30 + 70)	39	M = 16 (+YA 1) F = 19 (+YA 4)	40.0	
R_4_	128	3 different days (Mx. = 55; Mi. = 21)	31	M = 17 (+YA 8) F = 28 (+YA 5)	45.3	Copious exudates in gastric mucosa, no lesion revealed
R_5_	546	10 different days (Mx. = 172; Mi. = 10)	41	Bunches of light pinkish worms (M + F) recovered	—	
GP_1_	130	Single dose	108	—	—	—
GP_2_	50	3 different days (Mx. = 28; Mi. = 8)	41	M = 2 F = Nil	4.0	No lesion noted in the mucosa

*Ph* *ys* *oc* *ep* *ha* *lu* *s* *sexalatus*						
R_1_	279	9 different days (Mx. = 80; Mi. = 6)	45	Two L_3_ recovered from multiple nodules on the gastric wall	—	Many nodular lesions showed cut sections of larvae. Several encapsulated larvae detected in the cut sections of gastric wall.
R_2_	87	10 different days (Mx. = 21; Mi. = 3)	48	One L_3_ recovered from the omentum close to ventral lobe of liver	—	
R_3_	223	11 different days (Mx. = 72; Mi. = 50)	53	No larvae recovered	—	Several encapsulated larvae found in the cut sections of stomach wall.

*Sp* *i* *ro* *c* *er* *ca* *lupi*						
R_1_	123	12 different days (Mx. = 52; Mi. = 1)	59			Encystment on the gastric wall; 4 nodular lesions on the intima 4–8 mm from the bulbous aorta.
R_2_	90	4 different days (Mx. = 54; Mi. = 7)	37 (died on the previous night)	14 YA recovered from the intima; may cut in the lesioned patches in stomach wall (L_3_) or aorta (L_4_)	—	Internal haemorrhage detected on postmortem suspected either from rupture of minute vessels or wall of aorta.

*R: rabbit; GP: guinea pig; DPI: days postinfection; M: male, F: female, A: adult; YA: young adult; L_3_/L_4_: third-or fourth-stage larvae, Mx: maximum; Mi: minimum; —: not counted/no recovery.

**Table 2:** *Toxocara canis*: host related effect between large, medium, and small types.

Type	Length	Diameter	Volume/area
Large (*n* = 2)	17.45 ± 0.65^a∗∗^	2.2 ± 0.00^a∗∗^	38.39 ± 1.43^a∗∗^
Medium (*n* = 4)	11.30 ± 0.49^b∗∗^	1.85 ± 0.12^a∗∗^	20.93 ± 1.73^b∗∗^
Small (*n* = 8)	7.01 ± 0.77^c∗∗^	1.10 ± 0.07^b∗∗^	7.80 ± 1.06^c∗∗^
Overall (*n* = 14)	9.73 ± 1.11	1.10 ± 0.07	15.92 ± 3.09

All specimens in Tables 2 and 3 are gravid females; length expressed in cms and diameter in mm. Means within a column with different superscript differing significantly (**P < 0.01).

**Table 3:** *Toxocara canis*: Host related effect between local (Ludhiana) versus passage (Assam type).

Type	Length	Diameter	Area
Ludhiana *n* = 2	7.65 ± 0.25	1.50 ± 0.00	11.47 ± 0.38
Passage *n* = 2	6.70 ± 0.40	0.90 ± 0.10	6.07 ± 1.03
*F* value	4.056	36.00*	24.31*
Overall *n* = 4	7.12 ± 0.34	1.20 ± 0.18	8.77 ± 1.62

Significance level *P* < 0.05.

**Table 4:** Development of *Toxocara canis* in pups/dogs (mouse passage of L_2_).

Age in months and sex	Necropsy (DPI)	Recovery of adults		Percentage
		Male	Female	
3 female	58	33	35	22.6
2.5 female	58	03	07	3.3
3 female	57	46	42	29.3
2.5 female	89	02	10	4.0
2 male	26 (died)	—	—	—
3 male	45	37	47	28.0
3 male	58	—	—	—
2.5 female	47	07	09	5.3

			Mean	17.7
